# Optimization of the heme biosynthesis pathway for the production of 5-aminolevulinic acid in *Escherichia coli*

**DOI:** 10.1038/srep08584

**Published:** 2015-02-26

**Authors:** Junli Zhang, Zhen Kang, Jian Chen, Guocheng Du

**Affiliations:** 1 The Key Laboratory of Industrial Biotechnology, Ministry of Education, Jiangnan University, Wuxi 214122, China; 2 School of Biotechnology, Jiangnan University, Wuxi 214122, China; 3 Synergetic Innovation Center of Food Safety and Nutrition, Jiangnan University, Wuxi, Jiangsu 214122, China; 4 The Key Laboratory of Carbohydrate Chemistry and Biotechnology, Ministry of Education, Jiangnan University, Wuxi 214122, China

## Abstract

5-Aminolevulinic acid (ALA), the committed intermediate of the heme biosynthesis pathway, shows significant promise for cancer treatment. Here, we identified that in addition to *hemA* and *hemL*, *hemB*, *hemD*, *hemF*, *hemG* and *hemH* are also the major regulatory targets of the heme biosynthesis pathway. Interestingly, up-regulation of *hemD* and *hemF* benefited ALA accumulation whereas overexpression of *hemB*, *hemG* and *hemH* diminished ALA accumulation. Accordingly, by combinatorial overexpression of the *hemA*, *hemL*, *hemD* and *hemF* with different copy-number plasmids, the titer of ALA was improved to 3.25 g l^−1^. Furthermore, in combination with transcriptional and enzymatic analysis, we demonstrated that ALA dehydratase (HemB) encoded by *hemB* is feedback inhibited by the downstream intermediate protoporphyrinogen IX. This work has great potential to be scaled-up for microbial production of ALA and provides new important insights into the regulatory mechanism of the heme biosynthesis pathway.

5-aAminolevulinic acid (ALA), a non-protein amino acid, is the first committed intermediate in the common tetrapyrrole pathway (Fig. 1) for synthesis of heme, chlorophyll, cytochrome and vitamin B_12_^1^. In nature, there exist two known alternate routes by which this committed intermediate is generated^2^,^3^. One route is the C4 pathway (Shemin pathway), which involves the condensation of succinyl-CoA and glycine to ALA by ALA synthase (ALAS). The C4 pathway is restricted to mammals, fungi and purple nonsulfur bacteria^4^. The second route is the C5 pathway^5^, which involves three enzymatic reactions resulting in the biosynthesis of ALA from glutamate. The C5 pathway is active in most bacteria, all archaea and plants (Fig. 1).

ALA has recently attracted much attention for its great potential applications in the fields of medicine (tumor-localizing and cancer photodynamic therapy)^6^,^7^,^8^ and agriculture (biodegradable herbicides, plant growth regulators and insecticides)^1^,^2^. Since chemical synthesis of ALA is complicated and low-yielding, studies focusing on alternative microbial production of ALA from renewable and inexpensive sources has received much attention^1^. In this regard, many native microbes including photosynthetic bacteria (*Rhodobacter sphaeroides*) have been isolated and randomly mutated to produce ALA^2^,^9^,^10^. Although the titer of ALA has been significantly improved, its application was confined due to many disadvantages, such as the requirement of light illumination and poor cell growth. Consequently, intensive research has been concentrated on construction of recombinant *Escherichia coli* cell factories for enzyme-transformation of ALA by heterologous expression of different ALAS genes^11^,^12^. Notably, by applying a full factorial design model, the production of ALA was significantly increased to 5.20 g l^−1^ (39 mM)^13^. In addition, a highest titer of ALA was achieved (7.30 g l^−1^, 56 mM) after optimization of ALAS expression and cultivation process^14^,^15^,^16^. However, application of the complex medium (LB), artificial addition of the precursors glycine and succinate and the complicated cultivation process would be unbeneficial to industrial production.

Alternatively, from metabolic engineering point of view, Kang et al. recently engineered the C5 pathway by co-overexpression of a mutant *hemA*^17^,^18^,^19^,^20^ (encodes glutamyl-tRNA reductase, *Salmonella arizona*) and *hemL* (encodes glutamate-1-semialdehyde aminotransferase, *E. coli*) in *E. coli* and successfully increased ALA accumulation to 2.01 g l^−1^. After further overexpression of *rhtA* (encodes a membrane protein for threonine and homoserine exporting, *E. coli*) and optimization of minimal medium composition and cultivation process, the titer of ALA was improved to 4.13 g l^−1^ in batch-fermentation with glucose as the sole carbon source. As an important precursor, the biosynthesis of ALA is tightly regulated by the end product heme. In addition, the regulation mechanism of the heme biosynthesis pathway is more complex than expected^21^. Although the heme biosynthesis pathway enzymes have been well studied^3^,^22^,^23^, limited information on its regulation mechanism is available^3^.

In the present work, we systematically investigated the effect of overexpression of the downstream genes (*hemB*, *hemC*, *hemD*, *hemE*, *hemF*, *hemG* and *hemH*) on ALA accumulation. Interestingly, we discovered that in addition to the upstream genes *hemA* and *hemL*, *hemB*, *hemD*, *hemF*, *hemG* and *hemH* are also the major regulatory targets. Specifically, up-regulation of *hemD* and *hemF* was beneficial to ALA accumulation whereas overexpression of *hemB*, *hemG* and *hemH* was adverse to ALA accumulation. Through combinatorial overexpression of *hemA*, *hemL*, *hemD* and *hemF*, the titer of ALA was increased to 3.25 g l^−1^. More importantly, in combination with transcriptional and enzymatic analysis, we demonstrated that ALA dehydratase (HemB) encoded by *hemB* is feedback inhibited by the downstream intermediate protoporphyrinogen IX.

## Results

### Up-regulation of *hemD* and *hemF* increased ALA accumulation

According to previous studies^24^,^25^, it has been accepted that the biosynthesis of ALA is the rate-limiting step for heme biosynthesis and is tightly regulated in organisms including *E. coli*. In order to investigate the effects of downstream genes of the heme synthesis pathway on ALA accumulation, we individually overexpressed *hemB*, *hemC*, *hemD*, *hemE*, *hemF*, *hemG* and *hemH* with a low-copy number vector pCDFDuet-1 in *E. coli*. Although the absolute production of ALA was low, distinct changes were observed with single overexpression of the above-mentioned genes (Fig. 2a). Up-regulation of *hemD* or *hemF* increased ALA accumulation while reverse results were obtained when overexpressing *hemB*, *hemG* or *hemH*. In contrast, no significant differences were detected after overexpression of *hemC* or *hemE*. These findings indicated that up-regulation of the downstream genes *hemD* or *hemF* has a positive correlation on ALA accumulation whereas up-regulation of *hemB*, *hemG* or *hemH* has a negative correlation. More importantly, the results also suggested that in addition to *hemA*, *hemL* and *hemF*^3^, *hemB*, *hemD*, *hemG* and *hemH* are also the main regulatory points of the heme biosynthesis pathway.

To further validate the above results and in view of ALA biosynthesis is tightly regulated in *E. coli*^17^, the upstream metabolic flux towards ALA was increased by co-overexpression of the rate-limiting enzymes HemA^s^ (a variant of HemA from *S*. *arizona*) and HemL (*E*. *coli*) in the above constructed recombinant strains. Compared to *E*. *coli* LA (*hemL* and *hemA*^s^) (862.5 mg l^−1^), *E*. *coli* LAD (*hemL*, *hemA*^s^ and *hemD*) and *E*. *coli* LAF (*hemL*, *hemA*^s^ and *hemF*) produced more ALA (951.6 mg l^−1^ and 1,138.2 mg l^−1^), respectively (Fig. 2b and Fig. 3a). In contrast, the recombinant strains *E*. *coli* LAG (*hemL*, *hemA*^s^ and *hemG*), *E*. *coli* LAH (*hemL*, *hemA*^s^ and *hemH*) and especially *E*. *coli* LAB (*hemL*, *hemA*^s^ and *hemB*) exhibited remarkably reduced ALA production which were 242.3 mg l^−1^, 580.6 mg l^−1^ and 39.2 mg l^−1^, respectively (Fig. 2b and Fig. 3a). Consistent with the above single expression results, no significant changes were observed when co-overexpressing *hemC* and *hemE*. The results confirmed that *hemD* and *hemF* are distinct from *hemB*, *hemG* and *hemH* in that they are beneficial to ALA production.

Additionally, overexpression of the above genes generated distinct effects on cell growth and glucose consumption. In particular, up-regulation of *hemB* resulted in reduced biomass (Fig. 3b) and glucose consumption (Fig. 3c), which is likely due to the accumulation of the harmful intermediate porphobilinogen (PBG) and its derivatives^12^ (Fig. 3d). Interestingly, overexpression of *hemG* resulted in more biomass coupled with a long lag phase (Fig. 3b and Supplementary Fig. S1), which is likely due to its direct involvement in energy generation^26^ and the toxic intermediate protoporphyrin IX^27^.

### Modular optimization of the committed enzymes to improve ALA production

**Figure 1:**
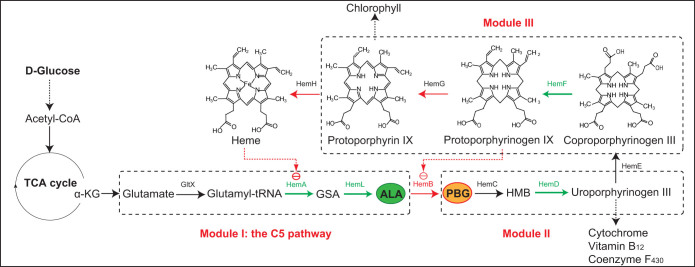
Illustration of the regulation mechanism of the heme pathway in *E. coli*. The pathway is divided into three modules (module I, module II and module III in the dotted box). The arrows in green and red represent the enzymes that are positive and negative to ALA accumulation, respectively. Dotted red arrows represent the feedback-inhibition. α-KG: α-ketoglutarate, GSA: glutamate-1-semialdehyde, ALA: 5-aminolevulinic acid, PBG: porphobilinogen, HMB: hydroxymethylbilane, GltX: glutamyl-tRNA synthetase, HemA: glutamyl-tRNA reductase, HemL: glutamate-1-semialdehyde aminotransferase, HemB: 5-aminolevulinic acid dehydratase, HemC: porphobilinogen deaminase, HemD: uroporphyrinogen III synthase, HemE: uroporphyrinogen decarboxylase, HemF: coproporphyrinogen III oxidase, HemG: protoporphyrin oxidase, HemH: ferrochelatase.

On the base of the above analysis, it could be speculated that simultaneous up-regulation of *hemD* and *hemF* would further enhance ALA production. To verify this speculation, the upstream genes (*hemA*^s^ and *hemL*) and the downstream genes (*hemD* and *hemF*) of the heme biosynthesis pathway were simultaneously overexpressed in *E. coli*. As expected, the resulting strain *E. coli* LADF harboring plasmids pACYCDuet-1-*hemL*-*hemA*^s^-*hemF* and pCDFDuet-1-*hemD* produced about 1,227.4 mg l^−1^ of ALA at 36 h (Fig. 4b), which was higher than that of the recombinant strains *E. coli* LAD (*hemL*, *hemA*^s^ and *hemD*) and *E. coli* LAF (*hemL*, *hemA*^s^ and *hemF*). To further fine-tune these four committed enzymes and improve the production of ALA, the compatible plasmids pRSFDuet-1 (high-copy number) and pETDuet-1 (medium-copy number) were used to investigate the impact of plasmid copy number on ALA production. According to the above results, the committed genes *hemA*^s^, *hemL*, *hemD* and *hemF* were distributed in three modules (Fig. 1). Specifically, due to HemA and HemL forming a tight complex, with a 1:1 ratio, to quickly catalyze glutamyl-tRNA to ALA^28^, the upstream genes *hemL* and *hemA*^s^ were contained within one operon and co-overexpressed with the downstream genes *hemD* and *hemF* as described in Fig. 4a. As we expected, the recombinant strains LADF-1, LADF-2 and LADF-3 with comparatively low-level overexpression of *hemL* and *hemA*^s^ resulted in dramatically decreased ALA accumulation which were 11.3 mg l^−1^, 211.7 mg l^−1^ and 12.1 mg l^−1^, respectively. In comparison, the recombinants LADF-4, LADF-5 and LADF-6 with high-level overexpression of *hemL* and *hemA*^s^ lead to much higher titer of ALA which were 1,202.8 mg l^−1^, 1,617.0 mg l^−1^ and 2,048.0 mg l^−1^, respectively. Furthermore, compared with the recombinant LADF-4 (RSF-*hemL*-*hemA*^s^, pBR322-*hemF*-*hemD*), LADF-6 (RSF-*hemL*-*hemA*^s^-*hemF*, pBR322-*hemD*) produced more ALA (2,048.0 mg l^−1^, Fig. 4b). The results suggested that high-level overexpression of the upstream genes *hemA*^s^ and *hemL* as well as the downstream gene *hemF*, and moderate overexpression of the downstream gene *hemD* are favorable to ALA accumulation.

To evaluate the capacity of the recombinant strain LADF-6 for the production of ALA, a scaled-up fermentation experiment was carried out in a 3 l bioreactor with *E. coli* LA as the control (Fig. 4c). Obviously, compared with *E. coli* LA, LADF-6 showed much bigger capacity for ALA accumulation (Fig. 4d). At 32 h, the titer of ALA was increased to 3.25 g l^−1^ with a final productivity of 0.102 g l^−1^ h^−1^ which was 1.79-fold of that of *E. coli* LA (1.82 g l^−1^). The results further demonstrated that up-regulation *hemD* and *hemF* is beneficial to ALA accumulation and rational up-regulation of the upstream genes (*hemA* and *hemL*) and the downstream genes (*hemD* and *hemF*) is a good strategy for efficient synthesis of ALA. Moreover, the results also confirmed that the regulatory mechanism of the heme biosynthesis pathway is complicated and needs to be uncovered.

### The heme biosynthesis pathway is tightly regulated at the transcriptional level

**Figure 2:**
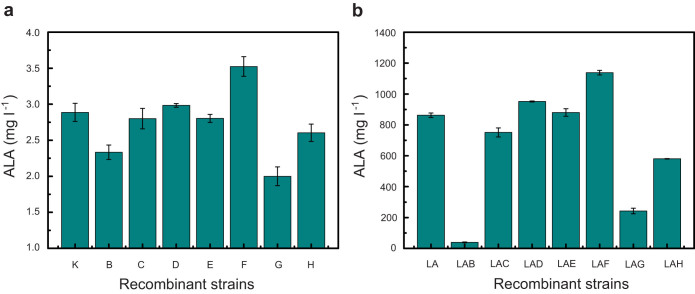
5-aminolevulinic acid production by recombinant strains. (a) The production of ALA by recombinant strains that individually overexpress *hemB*, *hemC*, *hemD*, *hemE*, *hemF*, *hemG* and *hemH*. (b) ALA production of recombinant strains that co-overexpress *hemL* and *hemA*^s^ with *hemB*, *hemC*, *hemD*, *hemE*, *hemF*, *hemG* and *hemH*, respectively.

To understand the underlying biology behind the different effects of overexpressing the above mentioned five key genes *hemB*, *hemD*, *hemF*, *hemG* and *hemH* on the heme biosynthesis pathway, we quantified the mRNA expression of these genes. As expected, single overexpression of these genes substantially increased the level of each respective mRNA transcript. Moreover, we observed that overexpression of *hemB*, *hemG* or *hemH* caused reduction of most of the pathway genes while overexpression of *hemD* resulted in significant increase of the all pathway genes (especially *gltX* and *hemA*) (Fig. 5), which are in agreement with ALA production (Fig. 2) and the results from the C4 pathway^29^. These findings suggested that *hemB*, *hemG*, *hemH* and *hemD* have central regulatory roles in the heme biosynthesis pathway and their transcriptional levels are tightly regulated.

**Figure 3:**
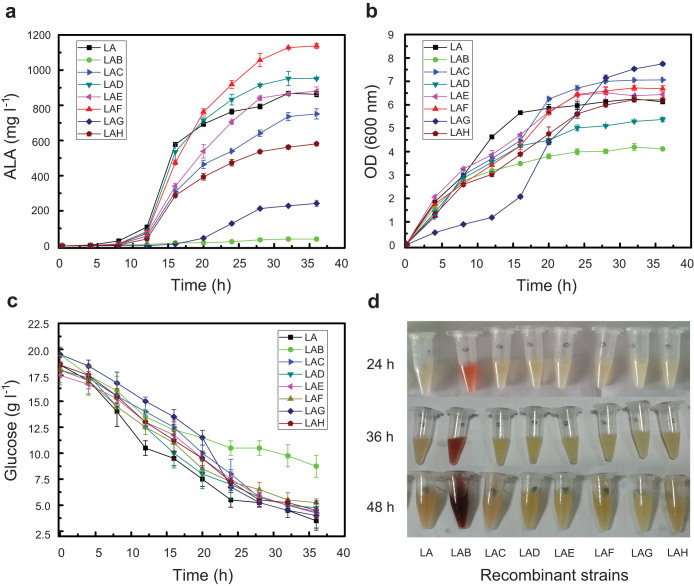
Time course of fermentation by recombinant strains. (a) ALA production. (b) Cell growth. (c) Glucose consumption. (d) The fermentation broth of the recombinant strains at 24 h, 36 h and 48 h.

Although ALA accumulation was significantly increased with overexpression of *hemF*, no obvious transcriptional alterations of the pathway genes were detected (Fig. 5), suggesting that up-regulation of *hemF* resulted in other effects but not transcriptional changes in pathway genes. Compared to *E*. *coli* LA (*hemL*, *hemA*^s^) and LAB (*hemL*, *hemA*^s^ and *hemB*), *E*. *coli* LAF (*hemL*, *hemA*^s^ and *hemF*) showed lighter color even with accumulation of more ALA (Fig. 3d). In addition, although up-regulation of *hemG* significantly decreased the transcriptional level of the genomic *hemA* gene, the recombinant strain *E*. *coli* LAG (*hemL*, *hemA*^s^ and *hemG*) harboring the plasmid-borne genes *hemL* and *hemA*^s^ still accumulated much less ALA compared with *E*. *coli* LA (*hemL*, *hemA*^s^). Taken together, we concluded that HemB is a key control node and might be feedback inhibited by the intermediate protoporphyrinogen IX which catalyzed from coproporphyrinogen III by coproporphyrinogen III oxidase (HemF, encoded by *hemF*) (Fig. 1).

### HemB is a feedback inhibitory node of the heme biosynthesis pathway

To further validate our speculation, the HemB activity of the recombinant strains *E*. *coli* LA (*hemL*, *hemA*^s^), *E*. *coli* LAF (*hemL*, *hemA*^s^ and *hemF*), *E*. *coli* LAG (*hemL*, *hemA*^s^ and *hemG*) and *E*. *coli* LAB (*hemL*, *hemA*^s^ and *hemB*) were directly examined (Table 1). Compared with *E*. *coli* LAB (52.04 U mg^−1^), *E*. *coli* LA exhibited much lower activity (6.97 U mg^−1^) which further confirmed that expression of HemB is tightly regulated to maintain a relatively low level in *E. coli*. Notably, *E*. *coli* LAF with overexpression of *hemF* showed an obvious decline of the HemB activity (3.34 U mg^−1^), which supported our speculation that HemB is feedback inhibited by the intermediate protoporphyrinogen IX (Fig. 1). Furthermore, *E*. *coli* LAG with overexpression of *hemG* gave rise to a substantial increase in HemB activity (22.21 U mg^−1^), which might be attributed to the direct activation by protoporphyrin IX or the weakened inhibition that caused by decreased protoporphyrinogen IX (Fig. 1).

To further confirm the above speculation and due to the unavailability of the intermediate protoporphyrinogen IX, we quantified HemB activity of the *E*. *coli* LAB strain extracts with addition of protoporphyrin IX *in vitro* to indirectly determine which of the two possibilities is correct. As shown in Table 1, activation of HemB following the addition of protoporphyrin IX was not detected. In combination with the above results from *E*. *coli* LAF, our speculation that HemB is a key regulatory node of the heme biosynthesis pathway and feedback inhibited by the intermediate protoporphyrinogen IX (Fig. 1) was demonstrated, which in turn provided the reason why high-level overexpression of *hemF* enhanced the production of ALA.

## Discussion

Because of its numerous potential applications in medicine and agriculture, market demand of the valuable compound ALA is rapidly rising. Over the past several years, much research has been dedicated to optimizing whole-cell catalytic synthesis of ALA^1^ by overexpression of ALAS. Although metabolic engineering has been widely used as a powerful tool for construction and optimization of the target pathways towards various valuable compounds^30^,^31^,^32^,^33^,^34^, few related studies have made significant contributions towards elucidating the regulatory mechanism of the heme biosynthesis pathway^1^,^2^,^35^,^36^ (Fig. 1), while the structure-function relationship for all the heme biosynthesis enzymes have been well understood^23^. Therefore, exploration of the heme synthesis pathway would not only be beneficial to rational engineering towards ALA production but also invaluable for uncovering regulatory mechanism of this highly conserved pathway.

Although the transcriptional model of *hemA* and *hemF* has been previously examined under different conditions^3^, the overall regulatory mechanism of heme biosynthesis pathway genes is still vague. In this study, we discovered that in addition to HemA, HemL and HemF^3^,^35^, HemB, HemD, HemG and HemH are also the key regulatory targets of the heme biosynthesis pathway. Overexpression of these genes showed significant effects on cell growth and metabolism (Fig. 3b and 3c) suggesting that they are tightly controlled under normal conditions. Recent studies have reported that *hemB* and *hemH* are negatively regulated by RyhB (an iron-associated small non-coding RNA) at the post-transcriptional level^37^. Moreover, by investigation at transcriptional and protein levels, we demonstrated that HemB is feedback inhibited by the intermediate protoporphyrinogen IX (Fig. 1), which suggest that regulation of heme biosynthesis pathway genes is far more complicated than imagined. In addition, Kang et al. previously discovered that overexpression of the native *gltX* gene (glutamyl-tRNA synthetase, GluRS) resulted in dramatic decrease of ALA^17^. Here, we also confirmed that overexpression of *gltX* and artificial addition of glutamate lead to reduced ALA accumulation and cell growth (Supplementary Fig. S2). The reason might be attributed to the synthesis of glutamyl-tRNA^Gln^ (harmful to cell growth) that catalyzed by the nonspecific GluRS^35^,^38^.

**Figure 4:**
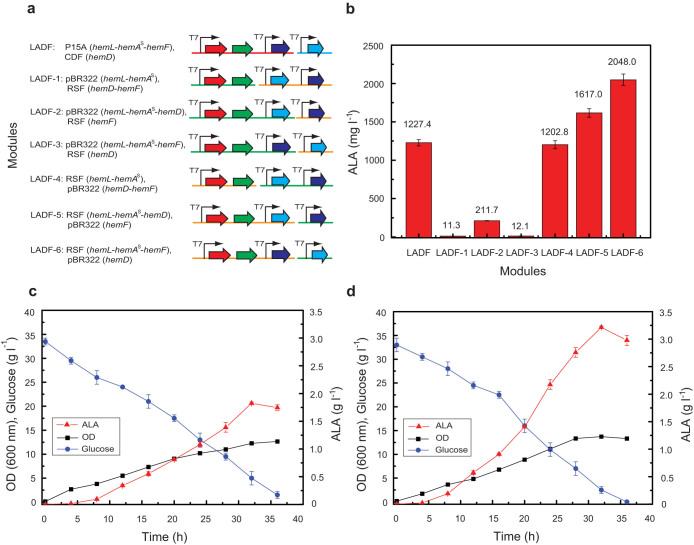
Modular optimization of ALA production by co-expression of *hemA*^s^, *hemL*, *hemD* and *hemF*. (a) Schematic representation of different combinations. The genes *hemL* (red arrow), *hemA*^s^ (green arrow), *hemD* (light blue arrow) and *hemF* (purple arrow) are assembled with the plasmids pACYCDuet-1 (red line), pCDFDuet-1 (blue line), pETDuet-1 (green line) and pRSFDuet-1 (orange line), respectively. (b) ALA production of recombinant strains with different gene combinations. (c) Batch fermentation of ALA by the engineered strain *E. coli* LA in a 3 l bioreactor. (d) Batch fermentation of ALA by the engineered strain LADF-6 in a 3 l bioreactor.

**Figure 5:**
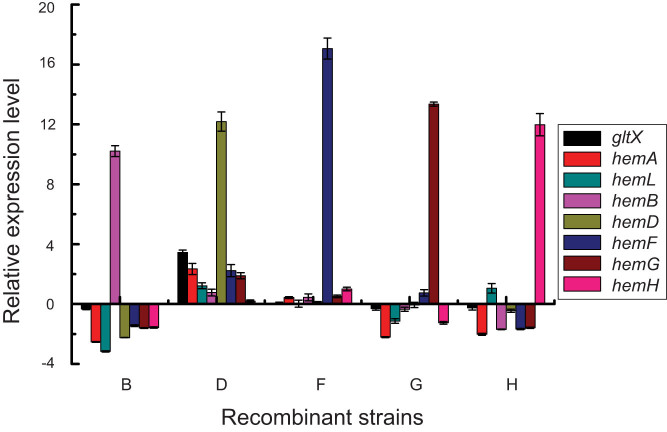
Alteration in pathway gene expression in the recombinant strains. Relative gene expression in recombinant strains was compared using *gapA* transcript levels for normalization. The control was the *E*. *coli* BL21 (DE3) harboring plasmid pCDFDuet-1. The results are on a logarithmic scale and the error bars represent one standard deviation of three independent experiments.

In the field of metabolic engineering, it was generally accepted that increase and balance of the objective synthetic pathways are the most critical parameters for high-yield production of the end-product^34^,^39^,^40^. For this purpose, many combinatorial optimization strategies have been developed and applied to assemble and regulate expression of the key genes^41^,^42^,^43^,^44^,^45^. In this study, after investigation and identification of the four positive key genes towards ALA, the heme biosynthesis pathway was further optimized with two compatible plasmids pRSFDuet-1 (high-copy number) and pETDuet-1 (medium-copy number) according to previous results. As we expected, the production of ALA was significantly increased (Fig. 4d) with an optimized combination of the increased flux towards ALA. One side, the results demonstrated that moderate expression of HemD is crucial to ALA accumulation since overexpression of HemD not only up-regulates the upstream genes (Fig. 5) but also draws more flux to downstream reactions from ALA (Fig. 1). Moreover, the results were consistent with our finding that HemB is a key regulatory point of the heme biosynthesis pathway and feedback inhibited by protoporphyrinogen IX. Previously, it has been reported that a *hemB* mutant *E*. *coli* strain failed to increase the production of ALA^46^ indicating that low activity of HemB is essential for cell growth and ALA accumulation. In comparison, the strategy of down-regulating HemB with overexpression of HemF was an alternative to improve ALA production.

In conclusion, through investigation of the heme biosynthesis pathway genes we discovered that *hemB*, *hemD*, *hemF*, *hemG* and *hemH* are also the main regulatory targets of the heme biosynthesis pathway. More importantly, overexpression of *hemD* and *hemF* increased the accumulation of the upstream intermediate ALA. By combinatorial overexpression of *hemA*, *hemL*, *hemD* and *hemF* with different copy-number plasmids, the titer of ALA was increased from 862.5 mg l^−1^ to 3.25 g l^−1^. Furthermore, in combination with transcriptional and enzymatic analysis, we demonstrated that HemB is feedback inhibited by the downstream intermediate protoporphyrinogen IX, which provides new important insights into the regulatory mechanism of the heme biosynthesis pathway. To further increase the production of ALA, comparative investigation of different *E. coli* hosts and overexpression of the ALA transporter RhtA^17^ should be available. In addition, dynamic regulation of HemB with synthetic regulatory elements or circuits^40^,^47^,^48^ should also be desirable for efficient production of ALA.

## Methods

### Strains and plasmids

Bacterial strains and plasmids used in this study are described in Supplementary Table S1 and Table S2, respectively. Specifically, *E*. *coli* BL21 (DE3) was used as host for gene expression due to the T7 RNA polymerase. *E*. *coli* JM109 was used for DNA manipulations and plasmids construction.

### Media and culture conditions

Luria-Bertani (LB) medium (g l^−1^) composed of tryptone 10.0, yeast extract 5.0 and NaCl 10.0 was used for the DNA manipulation process and seed cultures. Agar (2.0%) was added when a solid medium was required. The modified minimal medium (g l^−1^), which contains glucose 20.0, yeast extract 2.0, (NH_4_)_2_SO_4_ 16.0, KH_2_PO_4_ 3.0, Na_2_HPO_4_·12H_2_O 16.0, MgSO_4_·7H_2_O 1.0, MnSO_4_·H_2_O 0.01 was used for microbial production of ALA^17^. All recombinant strains were cultured at 37°C, 200 r min^−1^ and isopropyl-β-D-thiogalactopyranoside (IPTG) was initially added into the medium with a final concentration of 0.1 mM to induce the genes expression. Ampicillin (100 μg ml^−1^), chloramphenicol (20 μg ml^−1^), streptomycin (50 μg ml^−1^) or kanamycin (50 μg ml^−1^) was added to the medium for selection when necessary based on the harboring vectors.

The batch culture was performed in a 3 l fermentor (BilFlo 115, New Brunswick Scientific Co., Edison, NJ, USA). 2.0% inoculation of the seed culture was transferred into the fermentor with approximate 1.5 l medium after being cultured at 37°C for about 12 h. Chloramphenicol (20 μg ml^−1^) and streptomycin (50 μg ml^−1^) or ampicillin (100 μg ml^−1^) and kanamycin (50 μg ml^−1^), glucose (35.0 g l^−1^) and IPTG with a final concentration of 0.1 mM to induce the expression of genes were initially added to the medium. Agitation speed was 400 r min^−1^ and aeration rate was 1.0 vvm. The cultures were incubated at 37°C and pH was maintained at approximate 6.5 by adding 4.0 mol l^−1^ NaOH.

### Analytical procedures

All engineered *E*. *coli* strains were cultured in the modified minimal medium containing appropriate antibiotics. For growth studies, optical densities (OD) of the cell were measured at a wavelength of 600 nm with a UV-1,700 spectrophotometer (Shimadzu, Kyoto, Japan). Glucose concentration in the supernatant was detected using a glucose-glutamate analyzer SBA-40C (Biology Institute of Shandong Academy of Sciences, Jinan, China). The production of ALA was analyzed using the Modified Ehrlich's Reagent after the cultures were centrifuged^49^.

### Enzyme assays of HemB

For detecting HemB activity, cells were harvested after addition of IPTG following 8 h of culture by centrifugation for 10 min (10,000 r min^−1^, 4°C). Cells were washed twice with disodium hydrogen phosphate-citric acid buffer (pH 6.7), then they were resuspended in the above buffer and disrupted for 5 min using an ultrasonic oscillation (Sonics VCX750, amplitude 25%). After removing the cellular debris by centrifugation (10,000 r min^−1^, 4°C), the supernatant was analyzed for enzyme activity. Enzyme activity was quantified using the method described previously without the addition of dithiothreitol (DTT) to the reaction mixture^50^. HemB enzyme activity of one unit was defined as the amount of enzyme required to consume 1 pmol of ALA per min.

### Quantitative real-time PCR (qRT-PCR) analysis

Cultures used for RNA extraction were cultivated for approximately 5 h after the addition of 0.1 mM IPTG. Cell quantities corresponding to approximately 1.5–2.0 OD600 nm were harvested and frozen immediately at −80°C. Total RNA of all *E*. *coli* strains was extracted using the RNA Extraction Kit (Takara, Dalian, China) according to the manufacturer's instructions. The quantity and purity of the RNA was determined using a Nanodrop ND-1000 spectrophotometer (Thermo Scientific, Wilmington, DE, USA) by optical density measurements at 260 and 280 nm.

The RNA level was measured by qRT-PCR. Genes and their respective primer sequences that were used for qRT-PCR studies are listed in Supplementary Table S3. *gapA* encoding D-glyceraldehyde-3-phosphate dehydrogenase was selected as internal standard. The cDNA templates used for qRT-PCR were obtained by reverse transcribing mRNA transcripts using PrimeScript^TM^ RT-PCR Kit (Takara, Dalian, China). Gene expression analysis via qRT-PCR was carried out in a 96-well plate with a total reaction volume of approximately 20 μl using a SYBR® *Premix Ex Taq*^TM^ (Takara, Dalian, China) according to manufacturer's specifications. Reactions were performed with a LightCycler 480 II Real-time PCR instrument (Roche Applied Science, Mannheim, Germany).

## Author Contributions

Z.K. and J.Z. designed research; J.Z. performed research; Z.K. and J.Z. analyzed data; Z.K., J.Z., G.D. and J.C. wrote the paper; all authors reviewed the manuscript.

## Supplementary Material

Supplementary Information Supplementary Information

## Figures and Tables

**Table 1:** HemB enzyme activity in different recombinant strains

Recombinants	HemB activity (U mg^−1^)^a^	Protoporphyrin IX (mg l^−1^)^b^
*E*. *coli* LA	6.97 ± 0.26	-
*E*. *coli* LAF	3.34 ± 0.05	-
*E*. *coli* LAG	22.21 ± 0.42	-
*E*. *coli* LAB	52.04 ± 1.47	-
*E*. *coli* LAB	51.78 ± 1.50	5.0
*E*. *coli* LAB	51.70 ± 1.53	10.0

^a^Data are the means ± standard deviation (SD) from three parallel experiments.

^b^Intermediate protoporphyrin IX was added into the reaction system to study the effect on HemB activity.
