# Supplementary material for: Optimization of the heme biosynthesis pathway for the production of 5-aminolevulinic acid in Escherichia coli
Source: Sci Rep. 2015 Feb 26;5:8584. doi: 10.1038/srep08584 (PMC4341193; doi:10.1038/srep08584)
Supplement: Supplementary Information [file srep08584-s1.pdf]

Supplementary Information

Optimization of the heme biosynthesis pathway for the production of 5-aminolevulinic acid in *Escherichia coli*

Junli Zhang[1,2,3], Zhen Kang[1,2,3], Jian Chen[2,3] & Guocheng Du[2,4]

[1]The Key Laboratory of Industrial Biotechnology, Ministry of Education, Jiangnan University, Wuxi 214122, China; [2]School of Biotechnology, Jiangnan University, Wuxi 214122, China; [3]Synergetic Innovation Center of Food Safety and Nutrition, Jiangnan University, Wuxi, Jiangsu 214122, China; [4]The Key Laboratory of Carbohydrate Chemistry and Biotechnology, Ministry of Education, Jiangnan University, Wuxi 214122, China; Correspondence and requests for materials should be addressed to Z.K. (email: zkang@jiangnan.edu.cn) or G.C.D. (email: gcdu@jiangnan.edu.cn).

Supplementary Figure

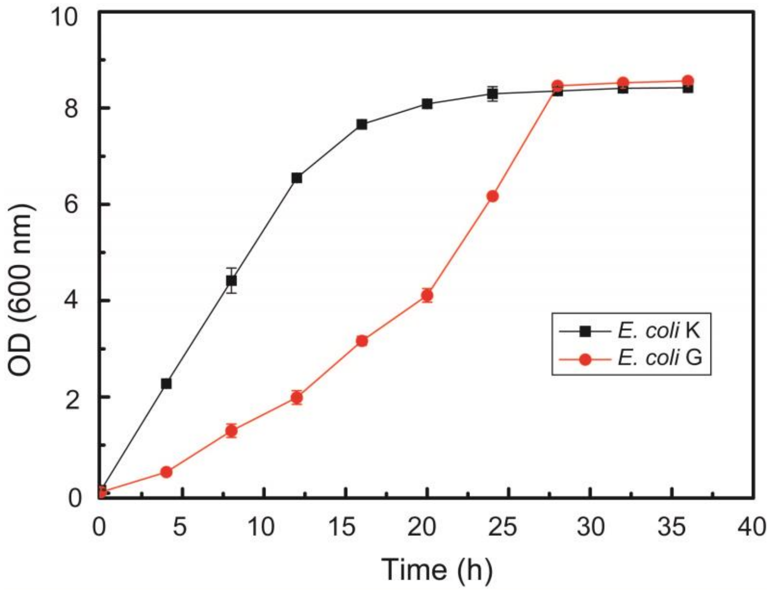

Supplementary Figure S1. Time course of the cell growth by recombinant strains *E. coli* K and *E. coli* G.

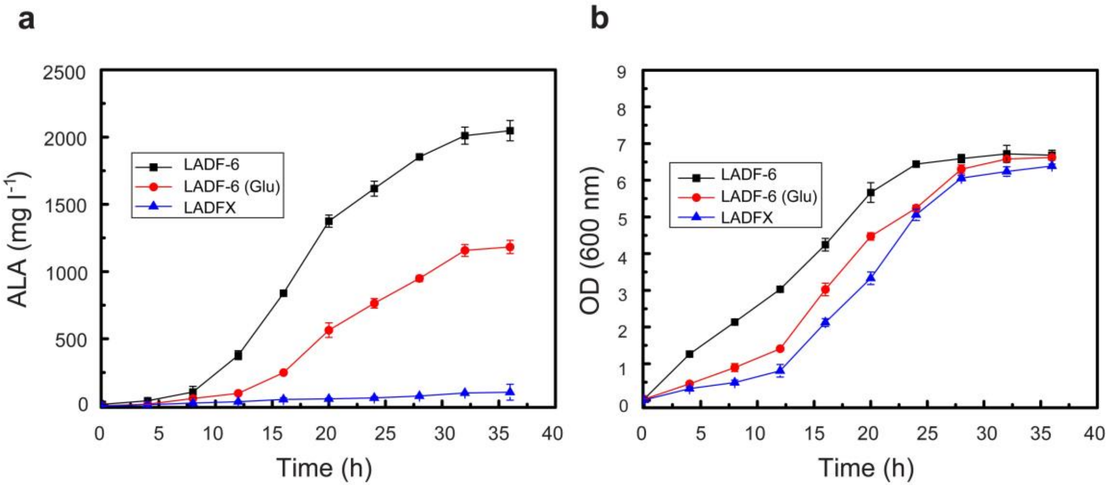

Supplementary Figure S2. The effect of glutamate and overexpression of *gltX* on the production of ALA and cell growth. (**a**) ALA production; (**b**) Cell growth. Black line represents recombinant *E. coli* LADF-6, red line represents recombinant *E. coli* LADF-6 with addition of glutamate and blue line represents recombinant *E. coli* LADFX with overexpression of *gltX*.

Supplementary Tables

Supplementary Table S1. Strains used in this study.

Strains	Relevant genotypes	References
Escherichia coli DH5α	F^-φ80 *dlacZ* ΔM15 Δ(*lacZYA-argF*)*U169 endA1 recA1 hsdR17* (rK$^-$ mK$^+$) *deoR thi*-1 *phoA supE44*λ^- *gyrA96 relA1*	Lab stock
E. coli JM109	*recA1 supE44 endA1 hsdR17 gyrA96 relA1 thi*-1 Δ(*lac-proAB*)/F' [*traD36proAB*+*lacIq E. coli lac*ZΔM15]	Lab stock
E. coli BL21 (DE3)	F^- *ompT hsdSB* (rB$^-$ mB$^-$) *gal dcm* (DE3)	Lab stock
E. coli K	*E. coli* BL21 (DE3) harboring pCDFDuet-1	This study
E. coli B	*E. coli* BL21 (DE3) harboring pCDFDuet-1-*hemB*	This study
E. coli C	*E. coli* BL21 (DE3) harboring pCDFDuet-1-*hemC*	This study
E. coli D	*E. coli* BL21 (DE3) harboring pCDFDuet-1-*hemD*	This study
E. coli E	*E. coli* BL21 (DE3) harboring pCDFDuet-1-*hemE*	This study
E. coli F	*E. coli* BL21 (DE3) harboring pCDFDuet-1-*hemF*	This study
E. coli G	*E. coli* BL21 (DE3) harboring pCDFDuet-1-*hemG*	This study
E. coli H	*E. coli* BL21 (DE3) harboring pCDFDuet-1-*hemH*	This study
E. coli LA	*E. coli* BL21 (DE3) harboring pACYCDuet-1-*hemL-hemA*^{s} and pCDFDuet-1	This study
E. coli LAB	*E. coli* BL21 (DE3) harboring pACYCDuet-1-*hemL-hemA*^{s} and pCDFDuet-1-*hemB*	This study
E. coli LAC	*E. coli* BL21 (DE3) harboring pACYCDuet-1-*hemL-hemA*^{s} and pCDFDuet-1-*hemC*	This study
E. coli LAD	*E. coli* BL21 (DE3) harboring pACYCDuet-1-*hemL-hemA*^{s} and pCDFDuet-1-*hemD*	This study
E. coli LAE	*E. coli* BL21 (DE3) harboring pACYCDuet-1-*hemL-hemA*^{s} and pCDFDuet-1-*hemE*	This study
E. coli LAF	*E. coli* BL21 (DE3) harboring pACYCDuet-1-*hemL-hemA*^{s} and pCDFDuet-1-*hemF*	This study
E. coli LAG	*E. coli* BL21 (DE3) harboring pACYCDuet-1-*hemL-hemA*^{s} and pCDFDuet-1-*hemG*	This study
E. coli LAH	*E. coli* BL21 (DE3) harboring pACYCDuet-1-*hemL-hemA*^{s} and pCDFDuet-1-*hemH*	This study
E. coli LADF	*E. coli* BL21 (DE3) harboring pACYCDuet-1-*hemL-hemA*^{s}-*hemF* and pCDFDuet-1-*hemD*	This study
E. coli LADF-1	*E. coli* BL21 (DE3) harboring pETDuet-1-*hemL-hemA*^{s} and pRSFDuet-1-*hemD-hemF*	This study
E. coli LADF-2	*E. coli* BL21 (DE3) harboring pETDuet-1-*hemL-hemA*^{s}-*hemD* and pRSFDuet-1-*hemF*	This study
E. coli LADF-3	*E. coli* BL21 (DE3) harboring pETDuet-1-*hemL-hemA*^{s}-*hemF* and pRSFDuet-1-*hemD*	This study
E. coli LADF-4	*E. coli* BL21 (DE3) harboring pRSFDuet-1-*hemL-hemA*^{s} and pETDuet-1-*hemD-hemF*	This study
E. coli LADF-5	*E. coli* BL21 (DE3) harboring pRSFDuet-1-*hemL-hemA*^{s}-*hemD* and pETDuet-1-*hemF*	This study
E. coli LADF-6	*E. coli* BL21 (DE3) harboring pRSFDuet-1-*hemL-hemA*^{s}-*hemF* and pETDuet-1-*hemD*	This study
E. coli LADFX	*E. coli* BL21 (DE3) harboring pRSFDuet-1-*hemL-hemA*^{s}-*hemF* and pETDuet-1-*hemD-gltX*	This study

Supplementary Table S2. Plasmids used in this study.

Plasmids	Relevant genotypes	References
pMD19 (Simple)	Cloning vector, Amp^R	Takara
pACYCDuet-1	Double T7 promoters, P15A ori, Cm^R	Novagen
pCDFDuet-1	Double T7 promoters, CDF13 ori, Sm^R	Novagen
pETDuet-1	Double T7 promoters, pBR322 ori, Amp^R	Novagen
pRSFDuet-1	Double T7 promoters, RSF ori, Kan^R	Novagen
pCDFDuet-1-*hemB*	pCDFDuet-1 containing *hemB*	This study
pCDFDuet-1-*hemC*	pCDFDuet-1 containing *hemC*	This study
pCDFDuet-1-*hemD*	pCDFDuet-1 containing *hemD*	This study
pCDFDuet-1-*hemE*	pCDFDuet-1 containing *hemE*	This study
pCDFDuet-1-*hemF*	pCDFDuet-1 containing *hemF*	This study
pCDFDuet-1-*hemG*	pCDFDuet-1 containing *hemG*	This study
pCDFDuet-1-*hemH*	pCDFDuet-1 containing *hemH*	This study
pACYCDuet-1-*hemL*-*hemA*^{s}	pACYCDuet-1 containing *hemL* and *hemA*^{s}	This study
pACYCDuet-1-*hemL*-*hemA*^{s}-*hemF*	pACYCDuet-1 containing *hemL*, *hemA*^{s} and *hemF*	This study
pETDuet-1-*hemL*-*hemA*^{s}	pETDuet-1 containing *hemL* and *hemA*^{s}	This study
pETDuet-1-*hemD*	pETDuet-1 containing *hemD*	This study
pETDuet-1-*hemF*	pETDuet-1 containing *hemF*	This study
pETDuet-1-*hemL*-*hemA*^{s}-*hemD*	pETDuet-1 containing *hemL*, *hemA*^{s} and *hemD*	This study
pETDuet-1-*hemL*-*hemA*^{s}-*hemF*	pETDuet-1 containing *hemL*, *hemA*^{s} and *hemF*	This study
pETDuet-1-*hemD*-*hemF*	pETDuet-1 containing *hemD* and *hemA*^{s}	This study
pRSFDuet-1-*hemL*-*hemA*^{s}	pRSFDuet-1 containing *hemL* and *hemA*^{s}	This study
pRSFDuet-1-*hemD*	pRSFDuet-1 containing *hemD*	This study
pRSFDuet-1-*hemF*	pRSFDuet-1 containing *hemF*	This study
pRSFDuet-1-*hemL*-*hemA*^{s}-*hemD*	pRSFDuet-1 containing *hemL*, *hemA*^{s} and *hemD*	This study
pRSFDuet-1-*hemL*-*hemA*^{s}-*hemF*	pRSFDuet-1 containing *hemL*, *hemA*^{s} and *hemF*	This study
pRSFDuet-1-*hemD*-*hemF*	pRSFDuet-1 containing *hemD* and *hemF*	This study
pETDuet-1-*hemD*-*gltX*	pETDuet-1 containing *hemD* and *gltX*	This study

Supplementary Table S3. Primers used for genetic manipulation in this study.

Primers	Sequence (5′-3′)*
hemL-hemA^s-F	CGCGGATCCATAAA**AGGAGGA**AAATATATGAGTAAGTCTGAA
hemL-hemA^s-R	TGCACTGCAGTTACTCCAGCCCGAGGCTG
hemB-F	CGCCATATGACAGACTTAATCCAACGCCC
hemB-R	CCGCTCGAGTTAACGCAGAATCTTCTTCTCAGC
hemC-F	CGCCATATGTTAGACAATGTTTTAAGAATTGCC
hemC-R	CCGCTCGAGTTATGCCGGGGCGTCT
hemD-F	CGCCATATGAGTATCCTTGTCACCCGCC
hemD-R	CCGCTCGAGTTATTGTAATGCCCGTAAAAGCG
hemE-F	CGCCATATGACCGAACTTAAAAACGATCGT
hemE-R	CCGCTCGAGTTAGCGGTGATACTGTTCAGACAGTC
hemF-F	CGCCATATGAAACCCGACGCACACC
hemF-R	CCGCTCGAGTTACACCCAATCCCTGACCTTAAT
hemG-F	CGCCATATGAAAACATTAATTCTTTTCTCAACA
hemG-R	CCGCTCGAGTTATTTCAGCGTCGGTTTGTC
hemH-F	CGCCATATGCGTCAGACTAAAACCGGTATC
hemH-R	CCGCTCGAGTTAGCGATACGCGGCAACA
hemD (2)-F	CGCGGATCCATAAA**AGGAGGA**AAATATATGAGTATCCTTGTCACCCGCC
hemD (2)-R	TGCACTGCAGTTATTGTAATGCCCGTAAAAGCG
gltX-F	CGCCATATGAAAATCAAAACTCGCTTCG
gltX-R	CCGCTCGAGTTACTGCTGATTTTCGCGTTC
Primers for qRT-PCR	
gapA (RT)-F	GTTCACGCTACTACCGCTAC
gapA (RT)-R	CATACCAGTCAGTTTGCCATTC
gltX (RT)-F	TGAAAGAGATGGCACAGAGC
gltX (RT)-R	GCGGTCCAGTCAGTAATCG
hemA (RT)-F	CCAGGCAGAGCAAGTTCG
hemA (RT)-R	ATTCAGGCGTTCGTTATCCC
hemL (RT)-F	TGGTCGTCGTGATGTAATGG
hemL (RT)-R	GCTTCTTCTGCCGCTTCC
hemB (RT)-F	GAGACAACACTTAGCCTTAACG
hemB (RT)-R	AGTCATCACGGAACGAATACC
hemD (RT)-F	TATCGTGAGCACTGGTTACTAC
hemD (RT)-R	CATCGTTGTCAGCGTTATCG
hemF (RT)-F	ATGAAACCCGACGCACAC
hemF (RT)-R	ACCACCATTACGCAACACC
hemG (RT)-F	AACAAGGGACGGACAAACG
hemG (RT)-R	GCGAATAGAAGCACCAATGAC
hemH (RT)-F	ACGCCGATAACCACGATTAC
hemH (RT)-R	GGAAGCCAGTTCACGAGTC

*Restriction sites used for cloning are underlined, and ribosome binding sites are in bold.

Supplementary Methods

DNA manipulations

The oligonucleotides synthesized by Sangon Biotech (Shanghai, China) and used for PCR amplification are listed in Supplementary Table S3. Genomic DNA of *E. coli* DH5α was used as a template for gene amplification. The PCR products purified with DNA Gel Extraction Kit (Thermo Fisher Scientific, America) were subcloned into a T-vector (pMD19, Takara, Dalian, China), and sequenced for verification (Sangon Biotech, Shanghai, China). Then the recombinant vectors were transformed into *E. coli* JM109 for the DNA manipulations and *E. coli* BL21 (DE3) for ALA production.

All plasmids constructed in this study are listed in Supplementary Table S2. Modular system was designed for investigation of regulatory mechanism and ALA production in *E. coli*. This system is based on four compatible plasmids belonging to different incompatibility groups, pACYCDuet-1, pCDFDuet-1, pETDuet-1, and pRSFDuet-1. Given that HemA and HemL are rate-limiting enzymes in the C5 pathway for ALA biosynthesis[17], a mutant *hemA* (named *hemA*^{s}) and *hemL* were amplified together and inserted into the pACYCDuet-1 vector using *Bam*HI and *Pst*I restriction sites, yielding the recombinant plasmid pACYCDuet-1-*hemL*-*hemA*^{s}. As pACYCDuet-1 contains an *Nco*I restriction sites, a base A and termination codon (TAA) was added after *Bam*HI site to avoid the frameshift mutation. And in order to enhance the translation efficiency, a sequence of ribosome binding site was added before the initial codon (ATG). Meanwhile, *hemB*, *hemC*, *hemD*, *hemE*, *hemF*, *hemG* and *hemH* were amplified using genomic

DNA of *E. coli* DH5α as template and inserted pCDFDuet-1 vector using *Nde*I and *Xho*I restriction sites, respectively. In addition, *hemF* was digested with *Nde*I and *Xho*I restriction enzymes and connected with pACYCDuet-1-*hemL*-*hemA*^{s}, which was cut with the same restriction enzymes, yielding the recombinant plasmid pACYCDuet-1-*hemL*-*hemA*^{s}-*hemF*. Each gene was placed under control of an induced T7 promoter.

Furthermore, pETDuet-1 and pRSFDuet-1 as a medium and a high copy number plasmids were used to assemble and optimize the expression of *hemA*^{s}, *hemL*, *hemD* and *hemF*. Similar to the above, plasmids pETDuet-1-*hemL*-*hemA*^{s}, pRSFDuet-1-*hemD*-*hemF*, pETDuet-1-*hemL*-*hemA*^{s}-*hemD*, pRSFDuet-1-*hemF*, pETDuet-1-*hemL*-*hemA*^{s}-*hemF*, pRSFDuet-1-*hemD*, pRSFDuet-1-*hemL*-*hemA*^{s}, pETDuet-1-*hemD*-*hemF*, pRSFDuet-1-*hemL*-*hemA*^{s}-*hemD*, pETDuet-1-*hemF*, pRSFDuet-1-*hemL*-*hemA*^{s}-*hemF* and pETDuet-1-*hemD* were constructed with the same restriction enzymes sites. Among them, *hemD* gene in pRSFDuet-1-*hemD*-*hemF* and pETDuet-1-*hemD*-*hemF* was amplified with the primers *hemD* (2)-F and *hemD* (2)-R. In addition, *gltX* was amplified using genomic DNA of *E. coli* DH5α as template and inserted to plasmid pETDuet-1-*hemD* with restriction sites *Nde*I and *Xho*I, yielding the plasmid pETDuet-1-*hemD*-*gltX*.
